# Impact of the Koala Project on clinical outcomes of preterm infants in two maternity hospitals

**DOI:** 10.1590/1984-0462/2024/42/2022106

**Published:** 2023-07-10

**Authors:** Lorena Dias Dantas, Ricardo Queiroz Gurgel, Verena Pimenta Santos, Debora Fontes Leite, Ikaro Daniel de Carvalho Barreto

**Affiliations:** a Universidade Federal de Sergipe, Aracaju, SE, Brazil.; b Universidade Tiradentes, Aracaju, SE, Brazil.; c Universidade Federal de Pernambuco, Recife, PE, Brazil.

**Keywords:** Prematurity, Oxygen, Retinopathy of prematurity, Bronchopulmonary dysplasia, Enterocolitis necrotizing, Deaths, Prematuridade, Oxigênio, Retinopatia da prematuridade, Displasia broncopulmonar, Enterocolite necrosante, Mortes

## Abstract

**Objective:**

To describe the impact of the Koala project (Actively Controlling Target Oxygen) on clinical outcomes in patients born with less than 36 weeks of gestation, in two maternity hospitals, comparing before and after the strategy implementation.

**Methods:**

This is an intervention study with 100 preterm infants with gestational age ≤36 weeks, who used oxygen in two maternity hospitals between January 2020 and August 2021. One of the hospitals was a private institution and the other was philanthropic. The goal for the target oxygen saturation with this project was 91–95%. Comparisons between the two stages (before and after the implementation of the project) were made evaluating the outcomes of retinopathy of prematurity, bronchopulmonary dysplasia, necrotizing enterocolitis, and deaths. The continuous variables were described using mean, median, standard deviation and interquartile interval. The significance level adopted was 5% and the software used was R Core Team 2021 (version 4.1.0).

**Results:**

After oxygen control use according to the Koala protocol, there was a significant reduction in the cases of retinopathy of prematurity (p<0.001) and bronchopulmonary dysplasia (p<0.001). There were no deaths in the second stage, and there was a non-significant increase in the absolute number of necrotizing enterocolitis cases.

**Conclusions:**

The Koala project seems to be an effective and feasible strategy to reduce adverse situations in the management of premature children, but research with a greater sample is needed.

## RESUMO

**Objetivo:**

Descrever o impacto do projeto Coala (Controle Ativo de Oxigênio Alvo) nos desfechos clínicos em pacientes nascidos com menos de 36 semanas de gestação, em duas maternidades, comparando antes e depois da implementação da estratégia.

**Métodos:**

Trata-se de um estudo de intervenção com cem prematuros vivos, com idade gestacional ≤36 semanas, que utilizaram oxigênio em duas maternidades entre janeiro de 2020 e agosto de 2021. A meta para a saturação de oxigênio alvo com este projeto foi de 91–95%. Comparações entre as duas etapas (antes e depois da implantação do projeto) foram feitas avaliando os desfechos de retinopatia da prematuridade, displasia broncopulmonar, enterocolite necrosante e óbitos. As variáveis contínuas foram descritas por meio de média, mediana, desvio padrão e intervalo interquartil. O nível de significância adotado foi de 5% e o *software* empregado foi o R Core Team 2021 (versão 4.1.0).

**Resultados:**

Observou-se que, após o uso de controle de oxigênio segundo o protocolo Coala, houve redução significativa nos casos de retinopatia da prematuridade (p<0,001) e displasia broncopulmonar (p<0,001). Não houve óbitos na segunda etapa e houve aumento não significativo no número absoluto de casos de enterocolite necrosante.

**Conclusões:**

O projeto Coala parece ser uma estratégia eficaz e viável para reduzir situações adversas no manejo de crianças prematuras, mas pesquisas com amostras maiores são necessárias.

## INTRODUCTION

Oxygen is the most used drug in neonatal intensive care units. Premature newborns are the group using it the most. They are very sensitive to its use as a supplementary form of treatment.^1^

Preterm infants who underwent hyperoxia for a prolonged time may develop bronchopulmonary dysplasia (BPD) and retinopathy of prematurity (ROP), among other conditions.^2^ The control of oxygen supply can reduce these clinical outcomes that are unfavorable to preterm infants.^2^

The Koala (Actively Controlling Target Oxygen) project aims to control the inspiratory fraction of oxygen according to a specific oxygen supply adjustment protocol to achieve a target saturation of 91–95% continuously. The project was first published on the Fernandes Figueira National Institute of Women, Children and Adolescent Health’s *website* ^3^ with the purpose of instituting the safe use of supplemental oxygen in premature newborns in Brazilian neonatal intensive care units.

This project is a mobilization strategy of the neonatal unit’s multidisciplinary team for target oxygen control. Since the uncontrolled use of oxygen leads to harmful consequences for premature newborns at any time during the neonatal period (delivery room, neonatal unit admission and post-discharge), the Koala project exclusively concerns the use of oxygen in preterm infants during hospitalization in the neonatal unit.^3^

Observing the importance of this clinical practice in neonatology, we implemented and evaluated the impact of the Koala project on premature newborns under 36 weeks, before and after the implementation of the strategy, in two maternity hospitals in Sergipe — one from the private sector and another a philanthropic institution with a large predominance of patients from the Brazilian Unified Health System (SUS).

This study aimed to describe the impact of the Koala project on clinical outcomes in patients born at less than 36 weeks of gestation, in two maternity hospitals, comparing before and after the implementation of the strategy.

## METHOD

This is an intervention study in which the impact of the Koala project implementation was evaluated in preterm infants of less than 36 weeks of gestational age who used supplemental oxygen, regarding the following outcomes: retinopathy of prematurity, BPD, necrotizing enterocolitis, and mortality. Gestational age was estimated by the pediatrician using the Capurro method (physical examination of the NB)^4^.

Data were collected from the newborn’s medical records by the first author of this manuscript, in two maternity hospitals located in Aracaju, capital of Sergipe, Northeast Brazil. One was a private maternity hospital and the other the largest maternity hospital in the state, a philanthropic institution with an extensive predominance of SUS patients.

We included 100 premature newborns from January 2020 to September 2021, with gestational age less than 36 weeks, who used oxygen at some point in the neonatal intensive care unit and who did not meet the exclusion criteria of the study: severe persistent pulmonary hypertension, congenital malformations incompatible with life, cyanogenic congenital heart disease, severe respiratory distress syndrome, severe perinatal asphyxia.

From January to July 2020, the database of the first stage was filled in. The patients were observed according to the protocol currently used in each hospital, and the oxygen supply was not systematically controlled in this first moment. There were no additional risks to the premature infant besides those inherent to prematurity.

In August and September 2020, multidisciplinary teams including nurses, nursing assistants, neonatologists and physical therapists were trained to implement the Koala protocol in the chosen maternity hospitals. From October 2020 to September 2021, after the implementation of the project, the database was filled in. The target oxygen control was defined for the clinical observation process that was already taking place according to the protocols of each intensive care unit of the hospitals.

Maternal and neonatal data of the 100 eligible children were observed, as well as their clinical evolution before and after the control of oxygen supply. After the intervention, the outcomes of BPD, necrotizing enterocolitis, ROP and death were compared. Oxygen saturation records were collected from pulse oximetry. The oxygen saturation data of each patient were recorded in a flash drive, with the information from the multiparameter monitor, or, in its absence, nursing records.

The Koala protocol implemented in the studied maternity hospitals was as follows:

- From 91 to 95%: do nothing.
- Above 95%: slow and gradual drops of 1 to 2% in oxygen supply, according to the visible response in pulse oximetry.
- From 88 to 91%: slow and gradual increases of 1 to 2% in oxygen supply, according to the visible response in pulse oximetry.
- Below 88%: report to the doctor/nurse.

It was necessary to make a few adjustments because the blenders available in both hospitals were graded every 5 FiO2 points. In both hospitals, then, the blenders were subjectively adjusted to one or two points, according to the patient’s saturation, which was an adaptation to the protocol.

Categorical variables were described through absolute frequency and relative percentage. The continuous variables were described through the median and interquartile interval. The hypothesis of independence between the categorical variables was tested using Fisher’s Exact and Pearson’s chi-square tests. The Likelihood Ratio Test from the log-linear model for the conditional independence hypothesis was used to evaluate clinical outcomes according to gestational age groups. The hypothesis of adherence of the continuous variables to the normal distribution was tested using the Shapiro-Wilk test. The continuous variables were described through the mean, median, standard deviation, and interquartile interval. Once unconfirmed, the median equality hypothesis was tested using the Mann-Whitney test. The significance level adopted was of 5% and the software used was R Core Team 2021 — version 4.1.0 (A Language and Environment for Statistical computing. R Foundation for Statistical Computing, Vienna).

The Research Ethics Committee of the University Hospital (HU)/Federal University of Sergipe authorized the study on September 9^th^ 2019 under the Certificate of Presentation for Ethical Appreciation — CAAE number 16326719.0.0000.5546.

## RESULTS

Table 1 shows the number of patients in the groups before the intervention, totaling 58, and after the implementation of the Koala project, totaling 42, in the private and philanthropic institutions, respectively.

**Table 1.** Characteristics of the population before and after the implementation of the Koala Project.

	Group		p-value
	Before	After	
Hospital n (%)			
1	24 (41.4)	21 (50)	0.421^F^
2	34 (58.6)	21 (50)	
Sex n (%)			
Female	21 (36.2)	23 (54.8)	0.071^F^
Male	37 (63.8)	19 (45.2)	
Gestational age (weeks) - median (IQR)	33 (31-35)	32 (30-33)	0.108^M^
Gestational age (weeks) - n (%)			
<28	4 (6.9)	6 (14.3)	0.137^Q^
28–31	18 (31)	12 (28.6)	
32–33	13 (22.4)	15 (35.7)	
34–36	23 (39.7)	9 (21.4)	
Types of delivery - n (%)			
Vaginal	29 (50)	20 (47.6)	0.842^F^
C-Section	29 (50)	22 (52.4)	
Maternal urinary infection - n (%)	12 (20.7)	7 (18.9)	1.000^F^
Chorioamnionitis - n (%)	5 (8.6)	0 (0)	0.075^F^
Rupture of membranes - n (%)	12 (20.7)	7 (17.1)	0.797^F^
Diabetes *mellitus* - n (%)	1 (1.7)	0 (0)	1.000^F^
Gestational diabetes - n (%)	3 (5.2)	1 (2.4)	0.640^F^
Gestational hypertension - n (%)	9 (15.5)	7 (17.1)	1.000^F^
HEELP syndrome - n (%)	2 (3.4)	2 (4.9)	1.000^F^
Preeclampsia - n (%)	4 (6.9)	0 (0)	0.140^F^
Septic shock. - n (%)	9 (15.5)	5 (11.9)	0.772^F^
Respiratory distress syndrome - n (%)	28 (48.3)	29 (74.4)	0.012^F^
Pneumonia - n (%)	4 (6.9)	3 (7.1)	1.000^F^
Transient tachypnea of newborn - n (%)	21 (36.2)	11 (26.2)	0.386^F^
Bronchopneumonia - n (%)	9 (15.5)	1 (2.4)	0.042^F^
Early neonatal infection - n (%)	40 (69)	31 (73.8)	0.660^F^
Early onset sepsis - n (%)	9 (15.5)	20 (47.6)	0.001^F^
Late onset sepsis - n (%)	2 (3.4)	4 (9.5)	0.235^F^
Neonatal jaundice - n (%)	45 (77.6)	31 (73.8)	0.813^F^
COVID 19 - n (%)	1 (1.7)	5 (12.2)	0.079^F^
% of SpO_2_ records			
<91% - median (IIQ)	1.9 (0–4.5)	4 (1.5–9.7)	0.008^M^
>95% - median (IIQ)	83.3 (70.4–93.5)	73.1 (56.2–87)	0.008^M^
<91% and SpO_2_>95% - median (IIQ)	87 (73.9–94.4)	76.6 (65.7–88.5)	0.010^M^
91% ≤SpO_2_ ≤95% - median (IIQ)	13 (5.6–26.1)	23.4 (11.5–34.3)	0.010^M^

n: absolute frequency; %: relative frequency percentage; IQR: Interquartile Range; F: Fisher Exact Test; M: Mann-Whitney Test; Q: Pearson’s Chi-Square Test; SpO_2_: pulse oximeter oxygen saturation.

Regarding the studied outcomes, there was a significant reduction in ROP and BPD (p<0.001), a reduction in the absolute number of deaths and an increase in necrotizing enterocolitis.

When it comes to the type of delivery, we observed opposite profiles in relation to the route of birth delivery, with more normal childbirths in the philanthropic hospital than in the private one. A similar percentage is observed in the institutions before and after the clinical intervention in relation to gestational ages.

Regarding maternal comorbidities, the most common was urinary infection, with an occurrence of 20.7% in the group before the intervention and 18.9% after. Other maternal diseases were evaluated before and after the intervention, as follows: premature rupture of membranes — 20.7 *vs*. 17.1%; gestational hypertensive disease — 15.5 *vs*. 17.1%; and preeclampsia — 6.9 *vs*. 0%.

Concerning neonatal comorbidities after birth, the most common was late neonatal jaundice, with an occurrence of 75% in the group before the intervention and 90.5% after, followed by early neonatal infection, with an occurrence of 69% in the group before the intervention and 73.8% after. Other neonatal diseases were early respiratory distress syndrome, with an occurrence of 48.3% in the group before the intervention and 74.4% after, and a significant p-value of 0.012; transient tachypnea of the newborn, with an occurrence of 36.2% in the group before the intervention and 26.2% after; early sepsis, with an occurrence of 8.3% in the group before the intervention and 66.7% after, and a significant p-value of 0.001; late sepsis, with a prevalence of 4.2% in the group before the intervention and 14.3% after; septic shock, with a prevalence of 15.5% in the group before the intervention and 11.9% after; bronchopneumonia, with a prevalence of 15.5% in the group before the intervention and 2.4% after, and a significant p-value of 0.042.

The occurrence of COVID-19 was also investigated in the groups studied, and an increase in the number of cases of this disease over the course of the pandemic was observed, with 1.7% in the case group and 12.2% in the control group. Patients were tested if the parents were COVID-19 positive.

Analyzing the oxygen saturation of preterm infants before and after the Koala project, we observed that the new protocol succeeded with a significant p-value of 0.010 for target saturation range between 91–95%.

Table 2 shows the occurrence of the outcomes studied according to specific gestational age intervals in both maternity hospitals, before and after the Koala project. There was a significant reduction in ROP and BPD and a reduction in absolute numbers of deaths. Regarding necrotizing enterocolitis, there was an increase in the absolute number, but without statistical significance.

**Table 2.** Clinical outcomes, including retinopathy of prematurity stages by gestational age, before and after the implementation of the Koala Project.

	Age group (weeks)				p-value
	<28 n (%)	28 a 31 n (%)	32 a 33 n (%)	34 a 36 n (%)	
Necrotizing enterocolitis					
Before	1 (25)	0	0	1 (4.3)	0.407
After	0 (0)	1 (8.3)	2 (13.3)	1 (11.1)	
Bronchopulmonary dysplasia					
Before	3 (75)	3 (16.7)	0	0	<0.001
After	2 (33.3)	1 (8.3)	1 (6.7)	0	
Retinopathy of prematurity stages					
Before					
1	2 (50)	0)	0	0	<0.001
2	1 (25)	15 (83.3)	9 (69.2)	7 (30.4)	
3	0	0	0	15 (65.2)	
4	1 (25)	3 (16.7)	4 (30.8)	1 (4.3)	
After					
1	3 (50)	5 (41.7)	1 (6.7)	0	0.002
2	0	4 (33.3)	4 (26.7)	2 (22.2)	
3	0	0 (0)	3 (20)	6 (66.7)	
4	3 (50)	3 (25)	7 (46.7)	1 (11.1)	
Deaths					
Before	1 (25)	1 (5.6)	1 (7.7)	1 (4.3)	0.951
After	0	0	0	0	

n: absolute frequency; %: relative frequency percentage. Likelihood Ratio Test from the log-linear model for the conditional independence hypothesis.

Lower gestational ages were more affected by BPD. Concerning gestational degrees and ages, when ROP was observed, it was found that the lower the gestational age, the higher the degree of retinopathy; therefore, the more severe the condition. In patients with gestational age of 32–33 weeks, there was an increase in the number of retinopathy in relation to grades 3 and 4. In the group of late preterm infants (34–36 weeks), there was a reduction in ROP in grades 2 and 3. As for ROP grade 4, there was an increase in the number of cases after the intervention of the Koala protocol, which may mean a type 2 error (small sample size).

## DISCUSSION

The present study showed the importance of controlling oxygen supply in premature patients by observing the reduction of the main clinical outcomes and mortality when this is done.

The patients of the maternity hospitals studied had different social profiles. At the private hospital, with private and health insurance patients, prenatal care was more complete. At the philanthropic hospital, patients had lower social statuses, and access to complete prenatal care was less regular. It is known that thorough prenatal care reduces neonatal mortality and the prematurity rate;^5^ however, in the present study, the results were similar in both maternity hospitals.

As a result of the implementation of the Koala protocol, target saturation was established between 91 and 95%, and a statistically significant p-value achieving this target range was observed, which demonstrates the project’s success. In this study, the following clinical outcomes were evaluated before and after the project: ROP, BPD, necrotizing enterocolitis and deaths.

In the NeOProM meta-analysis, with a total of 4,965 newborns younger than 28 weeks in the intensive care units of several countries, the saturation ranges of 85–89% to 91–95% were compared, as well as the outcomes in relation to them. It was observed that, for the lower saturation ranges (85–89%), there was a higher risk of death in 41% (14 to 74%). According to this meta-analysis, there was a reduction in deaths resulting from oxygen saturation between 91 and 95%, which the Koala project drew on to suggest this range as the target saturation to be followed, due to better outcomes for ROP, BPD and deaths.^2^

In the mentioned meta-analysis, necrotizing enterocolitis increased by 25% (5 to 49%) in children with saturations different from the target (lower).^2^ However, in the present study, there was an absolute increase of the disease in the target saturation range, opposing what was observed in the meta-analysis.

Another meta-analysis, the BOOST I Trial, observed preterm infants under 30 weeks of age, comparing the low (91–94%) and high (95–99%) saturation ranges. There was an increase in BPD at discharge for patients with a higher saturation rate, as well as greater need for home oxygen use.^6^ In our study, preterm infants with saturation between 91–95% presented a significant reduction (p<0.001) of cases of BPD.

Here, the occurrence of ROP in preterm infants under 36 weeks significantly decreased after the Koala project. In the meta-analysis, lower risk of ROP was observed: -26% (-8 to -41%) in preterm infants younger than 28 weeks, with the saturation range of 91–95% maintained and evaluated in this meta-analysis.^2^

Among the risk factors associated with ROP, oxygen use is the main one, with an occurrence of around 71.4%,^7^ associated with its development. The longer the exposure time, the greater the severity of the disease.^8^ A meta-analysis evaluated the adverse effects, such as increased incidence of ROP, which were associated with high levels of inspiratory fraction of oxygen in preterm infants.^1^

ROP demands a multidisciplinary approach to reduce adverse situations in the management of premature children and requires the involvement of a qualified and trained team, composed of neonatologists, nurses, nursing technicians and ophthalmologists. This is in order to implement an effective program of screening and prevention of blindness by ROP.^9^ In our study, the team training of nurses, nursing assistants, neonatologists and physical therapists was important for the success of the Koala project.

According to Davidson and Berkelhamer, about 40% of newborns in this age group develop BPD.^10^ It was observed, in this study, that the occurrence of BPD in patients under 28 weeks was reduced from 75% to 33.3% after the implementation of the Koala project, that is, occurrence of this disease was high before the project, but, after treatment, rates were closer to the literature.

We have not followed up our newborns to evaluate later mortality on the groups using different oxygen concentration limits, but Schmidt et al. observed no significant effect on the rate of death or disability at 18 months in extremely preterm infants, targeting 85 to 89% oxygen saturations when compared with 91 to 95%.^11^

The limitations of this study consisted of the lack of a blender with a one-point graduation to strictly apply the protocol, as well as the small number of patients, which could lead to a type 2 error. We were not able to blind the study. Further research needs to be carried out. Despite these limitations, the study is important for the scientific community because there are no comparative analyses before and after the implementation of the Koala Project in Brazil. It is also important to promote the implementation of oxygen supply control in maternity hospitals, which helps to protect against the emergence of comorbidities in preterm infants.

We observed positive impacts on the outcomes studied, which demonstrates the importance of the new protocol implemented in both maternity hospitals. Adherence to the protocol was satisfactory and the maternity hospitals continue to use it in their neonatal intensive care units, so that the percentage of patients who reach target saturation increases, improving the outcomes.

In conclusion, The Koala Project seems to be an effective and feasible strategy to reduce adverse situations in the management of premature infants and seems to be able to achieve the target oxygen saturation that should be systematically pursued in premature newborns with gestational age of less than 36 weeks. But it is not possible to determine what the optimal saturation targets are with this study design, so more studies are necessary.

## Data Availability

The database that originated the article is available with the corresponding author.
